# Host Genetic Factors Affect Susceptibility to Norovirus Infections in Burkina Faso

**DOI:** 10.1371/journal.pone.0069557

**Published:** 2013-07-19

**Authors:** Johan Nordgren, Léon W. Nitiema, Djeneba Ouermi, Jacques Simpore, Lennart Svensson

**Affiliations:** 1 Division of Molecular Virology, Department of Clinical and Experimental Medicine, Linköping University, Linköping, Sweden; 2 Centre de Recherche Biomoléculaire Pietro Annigoni Saint Camille CERBA/LABIOGENE, Université de Ouagadougou, Ouagadougou, Burkina Faso; 3 Centre de Recherche en Sciences Biologiques Alimentaires et Nutritionnelles (CRSBAN) Université de Ouagadougou, Ouagadougou, Burkina Faso; Columbia University, United States of America

## Abstract

Norovirus (NoV) constitutes the second most common viral pathogen causing pediatric diarrhea after rotavirus. In Africa, diarrhea is a major health problem in children, and yet few studies have been performed regarding NoV. The association of histo-blood group antigens (HBGA) and susceptibility to NoV infection is well established in Caucasian populations with non-secretors being resistant to many common NoV strains. No study regarding HBGA and NoV susceptibility has yet been performed in Africa. We collected 309 stool and 208 saliva samples from diarrheal children in Ouagadougou, Burkina Faso; May 2009 to March 2010. NoV was detected using real-time PCR, and genotyped by sequencing. Saliva samples were ABO, Lewis and secretor phenotyped using in house ELISA assays. NoV was detected in 12% (n = 37) of the samples. The genotype diversity was unusually large; overall the 37 positive samples belonged to 14 genotypes. Only children <2 years of age were NoV positive and the GII.4 NoVs were more frequent in the late dry season (Jan-May). NoV infections were observed less in children with the secretor-negative phenotype or blood group A (OR 0.18; p = 0.012 and OR 0.31; p = 0.054; respectively), with two non-secretors infected with genotypes GII.7 and GII.4 respectively. Lewis-negative (Le^a−b−^) children, representing 32% of the study population, were susceptible to GII, but were not infected with any NoV GI. GII.4 strains preferentially infected children with blood group B whereas secretor-positive children with blood group O were infected with the largest variety of genotypes. This is the first study identifying host genetic factors associated with susceptibility to NoV in an African population, and suggests that while the non-secretor phenotype provides protection; the Lewis b antigen is not necessary for GII infection.

## Introduction

Norovirus (NoV) is the most common cause of acute gastroenteritis worldwide, and it is estimated to cause 200, 000 deaths in children world-wide, mainly in developing countries [1]. It is now regarded as the second most common viral pathogen after rotavirus in pediatric diarrhea. With vaccines for rotavirus available, the importance of NoV in pediatric diarrhea is steadily increasing and several studies have indicated that NoV is a major cause of acute diarrhea in rotavirus vaccinated populations [2], [3].

NoV is a genus in the family *Caliciviridae* and exhibits high genetic diversity. The NoV genus can be divided into six genogroups (genogroup I [GI] to GVI). The GI and GII NoVs are the most common in humans and can be divided into at least 8 and 19 genotypes respectively [4]–[6]. While the epidemiological and clinical implications of the genotypes are not fully understood, reports indicate that GII.4 is the most prevalent genotype and also induces more severe symptoms as compared to other genotypes [7]–[10].

In sub-Saharan Africa, molecular epidemiology studies of NoV have been performed in countries such as South Africa, Cameroon, Botswana, Malawi and Ghana [11]–[15]. Most of these studies, however, have screened a limited number of samples during a short time frame, and the extent of infections and molecular epidemiology of NoV in Africa remains largely unknown.

Human NoV strains can bind to histo-blood group antigens (HBGAs) and demonstrate strain dependent infection patterns [9], [16]–[21]. The ABH and Lewis phenotypes are important for NoV infections, either as ligands or as restriction factors. Persons carrying ≥1 functional *FUT2* allele, and thus expressing α1,2 fucosyltransferase 2 are termed secretors and can express the A and B blood group antigens as well as H-type 1 and Lewis b (Le^b^) antigens on mucosa and in secretions. Homozygous individuals with nonsense mutations in the *FUT2* gene which gives rise to the non-secretor phenotype are almost completely protected from experimental and natural infections with NoV [16], [18]–[20]. The *FUT3* enzyme mediates the expression of Lewis antigens, either Lewis a (for non-secretors) or Lewis b (for secretors) with Lewis-negative individuals (inactive *FUT3* enzyme) unable the express either of these. Binding studies with virus-like particles [22], as well as studies with wild-type virus [17], [23]–[25], have shown that also non-secretors can be infected by certain NoV strains. Although many studies have been performed in Caucasian populations, the genetic polymorphisms that determines presence of HBGAs, particularly secretor and Lewis phenotypes, in African populations are different [26] and as of yet, no study has attempted to investigate how this could influence susceptibility to NoV in Africa. Moreover, most previous reports have studied susceptibility makers towards a limited number of NoV genotypes associated with defined outbreaks or in challenge studies [17]–[19], [23], [24].

In this study, we collected feces and saliva samples prospectively and investigated susceptibility markers towards a total of 14 different NoV genotypes. We observed that non-secretors as well as secretors with blood group A were less infected with NoV. Interestingly, we also found that NoV-infection was as common in Lewis-negative children representing 32% of the investigated, as in Lewis b positive children (54% of investigated) demonstrating that the Lewis b antigen is not required for symptomatic NoV infection for many genotypes.

## Materials and Methods

### Ethics Statement

The study protocol and consent procedure were approved by the Institutional Ethics Committee of Saint Camille Medical Center and the CERBA, Ouagadougou, Burkina Faso (2012CR/X-25/0135). Informed oral consent was given by parents or child guardians before samples were collected as described below. Oral consent was deemed more suitable for this study due to the high prevalence of illiteracy in the study population. After the consent was given, personal details, as well as, epidemiological data were recorded in a paper file.

### Study Population and Specimens

The study was conducted at the Saint Camille Medical Center CERBA/LABIOGENE in Ouagadougou, the capital of Burkina Faso. The samples were part of a larger epidemiological study conducted from May 2009 to March 2010 to identify the enteropathogens causing pediatric gastroenteritis [27], [28]. The stool and saliva specimens were collected from children less than 5 years of age, who presented with diarrheal illness. Stool specimens were collected in sterile containers at the microbiology laboratory in Saint Camille Medical Center CERBA/LABIOGENE. A 10% (wt/vol) stool suspension was prepared in PBS, and three aliquots were frozen at −20°C for further analysis.

### Clinical Assessment

The clinical information was obtained by reviewing the clinical records of the cases as described previously [28]. After informed consent was given by parents or child guardians, information regarding age, sex, place of residence, ethnicity and symptoms such as fever (≥38°C), nausea, vomiting, loss of appetite, duration and number of loose stools during the past 24 h, as well as information on dehydration status, nutrition status, and whether the children had been using antibiotic and antiparasitical medicine were registered. All children and stool specimens were clinically evaluated by general practitioners following a local adaption of the World Health Organization (WHO) strategy for diarrheal management [29]. Dehydration was classified into “severe dehydration,” “some dehydration,” and “no dehydration”, according to the WHO guidelines.

### Detection of Histo-blood Group Antigens in Saliva

The ABO histo-blood group phenotype of secretor-positive persons and the Lewis phenotype (Lewis a and Lewis b) were determined in 208 children by a saliva-based ELISA, essentially as described previously [17], [30]. Briefly ELISA plates (NUNC 96F Maxisorp; Thermo Fisher Scientific, Roskilde, Denmark) were coated with saliva, diluted 1∶500 in coating buffer (0.1 M carbonate–bicarbonate buffer, pH 9.6); plates were incubated for 2 h at 37°C followed by 4°C overnight: The following day the plats was incubated for 1.5 h at 37°C with antibodies α-A (ABO1 clone 9113D10), α-B (ABO2 clone 9621A8) (Diagast, Loos Cedex, France), α-Le^a^ (Seraclone, LE1 clone 78FR 2.3), and α-Le^b^ (Seraclone LE2 clones LM129-181 and 96 FR2.10) (Biotest AG, Dreieich, Germany). Antibodies were diluted 1∶5000 in phosphate-buffered saline with 5% fetal bovine serum (Invitrogen AB, Lidingö, Sweden) and 0.05% Tween 20 (Sigma-Aldrich). After 4 washes, horseradish peroxidase–conjugated goat anti-mouse IgG (heavy plus light chain) (Bio-Rad Laboratories, Hercules, CA, USA), diluted 1∶10 000, was added, and plates were incubated for another 1.5 h at 37°C. The reaction was developed using TMB (DakoCytomation, Carpinteria, CA, USA) and stopped by addition of 2 M H_2_SO_4_.

### Determination of Secretor Status using UEA-1 lectin ELISA Assay

In a subset of saliva samples (n = 38) the results from the HBGA assay was insufficient in order to establish secretor phenotype. For these samples, we used a lectin based ELISA assay specific for Fucα1-2Gal-R present in secretor, but not non-secretor, saliva. ELISA plates (NUNC 96F Maxisorp; Thermo Fisher Scientific, Roskilde, Denmark) were coated with saliva, diluted 1∶500 in coating buffer (0.1 M carbonate–bicarbonate buffer, pH 9.6); plates were incubated for 2 h at 37°C followed by 4°C overnight. The following day the plate was blocked for 1 h at 37°C with 3% BSA in PBS; followed by the addition of HRP-conjugated *Ulex europaeus* agglutinin (UEA-I, Sigma Aldrich, Sweden) diluted 1∶3200; and incubated for 1.5 hrs at 37°C. The reaction was developed using TMB (DakoCytomation, Carpinteria, CA, USA) and stopped by addition of 2 M H_2_SO_4_. Four established secretor positive saliva and 4 established secretor-negative saliva were always run in each plate as a control.

### Determination and Definition of Secretor and Lewis Status

A child was considered secretor-positive if Lewis B (phenotype [Le^a−b+^]), and/or A and/or B HBGAs were observed in saliva. Similarly a child was considered secretor-negative if Lewis A was observed in saliva (phenotype [Le^a+b−^]), and Lewis-negative if neither Lewis a nor Lewis b were present in saliva (phenotype [Le^a−b−^]). For a number (n = 38) of Lewis-negative children, secretor status was determined by UEA-1 lectin assay described above; since these could be either secretor-positive with blood group O or secretor-negatives. Three NoV-negative children plausibly exhibited weak-secretor phenotype (high levels of Lewis a and low levels of Lewis b in saliva), but in absence of DNA for genotype verification these were excluded from analysis.

### Viral RNA Extraction

Viral RNA extraction from stool suspensions (10% PBS [wt/vol]) was performed using the BioRobot M48 (Qiagen, Hilden, Germany) according to the manufacturer’s instructions and the RNA was stored at −80°C until further use.

### Reverse Transcription

Reverse transcription (RT) was carried out as described previously [7]. 28 µl of RNA was mixed with 2.5 µg of random hexadeoxynucleotides [pd(N)6] (GE Healthcare, Uppsala, Sweden and added to one RT-PCR bead (GE Healthcare, Uppsala, Sweden) with RNase-free water to a final volume of 50 µl. The RT reaction was carried out for 30 min at 42°C for cDNA synthesis.

### Norovirus Detection and Genogrouping by Real-time PCR

Norovirus was screened using a multiplex TaqMan real-time PCR modified from [31], [32] as described previously [33]. The primers used were NVG1f1b, NVG1rlux, NVG2flux1, COG2R [31], [32] with genogroup I specific probe [6FAM]AGATYGCGRTCYCCTGTCCA[BHQ1] and genogroup II specific probe [JOE]TGGGAGGGCGATCGCAATCT[BHQ1] (modified from [31] and targeting the ORF1-ORF2 junction. The real-time PCR program was performed on a Corbett Research Rotor-Gene 3000 (Corbett Research, Concorde, Australia) with the following cycling conditions. An initial denaturation step for 10 min on 95°C followed by 45 cycles of 95°C for 15 s and 56°C for 1 min with continuous fluorescence reading. Based on the wavelength of emitted light, it is possible to detect and determine norovirus GI and GII simultaneously [33].

### Nucleotide Sequencing

Nucleotide sequencing was performed by Macrogen Inc. (Seoul, South Korea). The sequencing reaction was based on BigDye chemistry, using the same primers as in the PCR reaction as sequencing primers.

### Sequence Analysis

Multiple sequence alignment of the obtained nucleotide sequences of the partial N-terminal and Shell (NS) region (nt 1-301, ORF2) was performed using the ClustalW algorithm with default parameters on the European Bioinformatics Institute server. Phylogenetic analysis of the aligned file was performed using MEGA software, version 5.0, based on the neighbour-joining method. Phylogenetic distances were measured by the Kimura two-parameter model. The statistical significance of the phylogenetic tree was supported by bootstrapping with 1,000 replicates.

### Accession Numbers for Nucleotide Sequences

The nt sequences for the NS region of the Burkina Faso NoV strains can be found in GenBank using accession numbers JX416387-JX416419. The accession numbers for genotype references sequences can be found in [4] : The accession numbers of other sequences used for phylogenetic analysis in this study and present in the phylogenetic trees are listed below:

**NoV GI:** WUG1 (AB081723); Incheon/152/2005 (HQ213833); Mie2001-U72/oy/G1 (AB097911); Hu/V1622/06/IND (AB447406); Hu/GI/C2-261208-1/Singapore/2008 (HM209185); Norovirus_water/GI.1/09-11-09b/2009/ZAF (HQ201655); Norovirus_Hu/GI.7/4349a/2008/ZAF (GU138767); Norovirus_Hu/GI/Seoul/stool/SK2/2008 (JF896119);

**NoV GII:** GII.4/New_Orleans1805/2009/USA (GU445325); GII/IPH2045-LIM008/2010/BE (JF697290); GII.1/3445/2008/ZAF (GU138772); GII.16/4349b/2008/ZAF (GU138769); V1628/06/IND (AB453773); CMH037/00/2000/THA (EU363859); GII.10/6952/2008/ZAF (GU724780); GII.17/C15b/Bonaberi/Cameroon (JF802507); GII/2005/8093/Chelyabinsk/RUS (FJ383845); GII/Nizhny_Novgorod/8252/2005/RUS (FJ264895); GII.13/Seoul/0947/2010 (HM635129); GII.6/Seoul/0925/2009/KOR (HM635127); GII.4/RotterdamP6D33/2006/NL_AB385638; GGII.4/Cairo6/2006/EGY (EU876886); GII.4/C17/Bonaberi/2009/Cameroon (JF802504): Hu/GII.4/Seoul/0949/2010/KOR (HM635187); GII.4/Seoul/0982/2010/KOR (HM635196). GGII.4/Cairo9/2007/EGY (EU876889); GII.4/A40/Limbe/2009/Cameroon (JF802500); GII.4/Terneuzen70/2006a (EF126964); GII.4/Nijmegen115/2006b/NL (EF126966).

### Statistical Analysis

Categorical data were analyzed using the χ^2^ test or Fisher exact test with 2-tailed significance. Unadjusted odds ratios (ORs) and 95% confidence intervals (CIs) were calculated using SPSS 19.0 (SPSS Inc., Chicago, IL, USA).

## Results

### Clinical Profiles of NoV Infections in Children in Burkina Faso

NoV infections were associated with fever (49%), vomiting (49%), and dehydration (62.4%) (Table 1). Nineteen (51%) of the NoV-positive samples were infected with other enteropathogens screened for in previous studies (rotavirus [n = 9], bacteria [n = 4] and parasites [n = 3], or mix of these [n = 3] [28]). We stratified clinical symptoms into “pure” NoV infections and NoV GII.4 infections (Table 1). The major differences comparing these groups was that NoV of genotype GII.4 were more associated with vomiting (70%), watery stool (90%), number of stools (80% ≥4 stools) and a longer duration of diarrhea (50% ≥4 days), as compared to non- GII.4 genotypes. No particular differences were seen comparing pure norovirus infections with NoV together with concomitant infections (Table 1).

**Table 1:** Clinical features of NoV-infected children with and without concomitant infections.

Number of NoV	Norovirus incl mix,n = 37(%)	Norovirus GII.4,n = 10 (%)	Norovirus non^2^GII.4, n = 25 (%)	Pure norovirus infection^3^,n = 18 (%)
Fever (≥38°C)	18 (49)	5 (50)	11 (44)	10 (56)
Duration of diarrhea (days)				
1–3	25 (68)	5 (50)	20 (80)	14 (78)
4–5	5 (14)	2 (20)	1 (4)	2 (11)
≥6	7 (19)	3 (30)	4 (16)	2 (11)
Vomiting	18 (49)	7 (70)	9 (36)	9 (50)
				
Loss of appetite	28 (76)	7 (70)	19 (76)	14 (78)
Mucus	24 (65)	7 (70)	16 (64)	11 (61)
Number of loose stools in the past 24 hours				
1–3	14 (38)	2 (20)	11 (44)	7 (39)
4–5	12 (32)	6 (60)	5 (20)	4 (22)
>6	11 (30)	2 (20)	9 (36)	7 (39)
Stools				
Watery	24 (65)	9 (90)	15 (60)	10 (56)
Loose	13 (35)	1 (10)	10 (40)	8 (44)
Dehydration status^1^				
Severe dehydration	2 (5.6)	0 (0)	2 (8)	0 (0)
Some dehydration	21 (58)	6 (67)	13 (52)	10 (59)
No dehydration	13 (36)	3 (33)	10 (40)	7 (41)

1 Dehydration status was not obtained for 1 of the NoV-positive (GII.4) children.

2 Two NoV positive could not be genotyped and were excluded from the analysis.

3 Enteropathogens screened: Rotavirus, *Shigella spp*, *Salmonella spp,* enteropathogenic *Escherichia coli*, *Giardia lamblia, Trichomonas intestinalis, Entamoeba histolytica/dispar.* Includes 4 GII.4 genotypes.

### Norovirus Infected Young Children and was not Associated with Malnutrition

Children <1 year of age were more frequently infected with NoV as compared to children >1 year of age (36% vs 11% p<0.05), with none of the children >2 years of age being NoV-positive (Table 2). No significant difference in sex was observed although NoV was slightly more prevalent in males (13%) as compared to females (10%). Malnutrition status was not associated with significant differences in NoV prevalence, although NoV was more frequently observed in undernourished children (15% vs 10%, Table 2).

**Table 2:** Epidemiological profile of pediatric norovirus diarrhea in children of ≤5 years of age from Ouagadougou, Burkina Faso.

		Total	No. of NoVpositive (%)n = 37
Age range (month)			
0–6	20		4 (20)
6–12	115		18 (16)
12–24	139		15 (11)
>24	35		0 (0)
Gender			
Male	164		22 (13)
Female	145		15 (10)
Underweight status^1^			
Z> −2 SD	131		19 (15)
−3 SD<Z>−2 SD	82		6 (7)
Z<−3 SD	94		11 (12)

1 Underweight status, weight-for-age *Z*-scores, data missing for 1 NoV (GII.4) positive child.

### Children with Non-secretor Phenotype were Less Infected with NoV and the Lewis b Antigen was not Required for Symptomatic GII NoV Infection

The saliva from 36 NoV-positive and 172 NoV-negative children with diarrhea was phenotyped regarding ABH and Lewis HBGAs. The distribution of Lewis phenotypes in the total phenotyped population (n = 208) was as follows; Le^a−b+^ (54%), Le^a−b−^ (32%) and Le^a+b−^ (14%); with 21% of the study population being non-secretors (Table 3).

**Table 3:** Host genetic factors and association to susceptibility to NoV infections in Burkina Faso.

Saliva phenotype		NoV positive 4(%)	NoV negative (%)	Odds ratio (95% c.i)	P value^5^
**Lewis phenotype**					
Le^a−b+^	n = 112	23 (21)	89 (79)	1.65 (0.78–3.47)	0.18
Le^a−b−1^	n = 66	12 (18)^2^	54 (82)	1.09 (0.51–2.35)	0.82
Le^a+b−^	n = 30	1 (3)	29 (97)	0.14 (0.019–1.07)	0.029
**ABO phenotype** 3					
A	n = 34	3 (9)	31(91)	0.31(0.088–1.08)	0.054
B	n = 55	14 (25)	41(75)	1.52 (0.70–3.31)	0.29
AB	n = 4	1 (25)	3 (75)	1.28 (0.13–12.74)	1.00^6^
O	n = 71	16 (23)	55 (77)	1.21 (0.57–2.59)	0.62
**Secretor status**					
Positive	n = 164	34 (21)	130 (79)	5.49 (1.27–24)	0.012
Negative	n = 44	2 (5)	42 (95)	0.18 (0.042–0.79)	0.012

1 21% and 79% of Lewis-negative children were non-secretors and secretors; respectively.

2 Including 1 non-secretor and 11 secretors.

3 Only determined for secretor-positive individuals.

4 Saliva was lacking for one NoV-positive child.

5 Chi square test with two-tailed significance.

6 Fisher exact test with two-tailed significance.

Similarly as reported from studies on Caucasian populations, NoV was observed less in children with the secretor-negative phenotype (OR 0.18 [0.042–0.79]; p = 0.012) (Table 3) with two non-secretors infected with NoV of genotypes GII.7 and GII.4, respectively. Most interestingly was however that NoV prevalence was similar in Le^a−b−^ individuals, representing 32% of the investigated compared to Le^a−b+^ individuals (OR 1.09 [0.51–2.35] vs 1.65 [0.78–3.47]) (Table 3). GI NoVs (n = 7) only infected Le^a−b+^ individuals, whereas GII NoVs (n = 29) was observed in all individuals irrespectively of Lewis status (Table 4 and 5), all suggesting that the Lewis b antigen is not required for infection of many GII genotypes, but possibly for most GI NoV infections observed in the study population. No statistical differences in NoV susceptibility and Lewis status were observed when comparing GII.4 and non-GII.4 genotypes (Table 4).

**Table 4:** Differences between NoV genogroups and genotypes regarding susceptibility patterns (%).

	Le^a−b+^	Le^a−b−^	Le^a+b−^	A^1^	B	O	AB
GI	7 (100)	0 (0)	0 (0)	0 (0)	1 (14)	6 (86)	0 (0)
GII	16 (55)	12 (41)	1 (3)	3 (11)	13 (48)	10 (37)	1 (4)
p-value^2^	0.034	0.07	1.0	1.0	0.20	0.035	1.0
GII.4	7 (70)	3 (30)	0 (0)	0 (0)	7 (78)	1 (11)	1 (11)
Non-GII.4	16 (64)	8 (32)	1 (4)	3 (13)	7 (29)	14 (58)	0 (0)
p-value	1.0	1.0	1.0	0.54	0.019	0.021	0.27

1 Blood groups were only determined for secretor-positive individuals.

2 Fisher exact test with two-tailed significance.

**Table 5:** Relationship between genogroups, genotypes, and HBGAs in NoV infected children in Burkina Faso.

		Blood groups^1^					Lewis phenotypes		
NoV type	n	O	A	B	AB	NA^2^	Le^a−b+^	Le^a−b−^	Le^a+b−^
Genogroup I	7	6	–	1	–	–	7	–	–
GI.1	1	1	–	–	–	–	1	–	–
GI.3	1	1	–	–	–	–	1	–	–
GI.6	1	1	–	–	–	–	1	–	–
GI.7	2	2	–	–	–	–	2	–	–
GI^2^	2	1	–	1	–	–	2	–	–
Genogroup II^4^	29	10	3	13	1	2	16	12	1
GII.1	1	1	–	–	–	–	1	–	–
GII.4	10	1	–	7	1	1	7	3	–
GII.6	3	1	2	–	–	–	1	2	–
GII.7	3	–	–	2	–	1	1	1	1
GII.8	1	–	–	1	–	–	1	–	–
GII.10	5	4	1	–	–	–	4	1	–
GII.14	1	1	–	–	–	–	–	1	–
GII.16	3	1	–	2	–	–	1	2	–
GII.17	1	–	–	1	–	–	–	1	–
GII^3^	1	1	–	–	–	–	–	1	–

1 Only determined for secretor-positive individuals.

2 Belonging to a yet undefined genotype (86.1% nt identity to most similar reference sequence).

3 Not genotyped.

4 Saliva was lacking for one NoV-positive child of genogroup II.

### Secretor-positive Children with Blood Group O were Infected with a Large Variety of Genotypes while Children with Blood Group A were Less Infected with NoV

The distribution of blood group phenotypes, determined in saliva only for secretor-positive children (n = 164) were; A (21%), B (34%), AB (2%) and O (43%) (Table 3). Children in the study population having blood group A were less infected with NoV infections (OR 0.31; p = 0.054), with the only NoV genotypes observed in children with blood group A were GII.6 (n = 2) and GII.10 (n = 1). Blood group O and B were not associated with an increase or decrease of susceptibility to NoV infections (Table 3).

We further stratified the results according to genogroups (GI and GII) and genotypes (GII.4 and non-GII.4 genotypes) (Table 4 and 5) in order to compare strain-dependent differences in HBGA susceptibility patterns. GI NoVs were significantly more associated with secretor-positive individuals having blood group O than GII NoVs (86% vs 37%, p<0.05) whereas GII NoVs were more often observed in secretor-positive individuals with blood group B as compared to GI NoVs (48% vs 14%, p = 0.2). When comparing genotype GII.4 with non-GII.4 genotypes we observed that GII.4 infected more secretor-positive children of blood group B than non-GII.4 strains (78% vs 29%, p<0.05) whereas non-GII.4 NoV strains had a preference for secretor-positive children of blood group O (58% vs 11%, p<0.05) (Table 4) in the study population.

In total 14 different NoV genotypes were observed in this study; 12 different genotypes were found in Le^a−b+^ children, 7 different genotypes in Le^a−b−^ children, and only 1 genotype in a Le^a+b–^ child (Table 5). Moreover, 11 different genotypes were found in secretor-positive children with blood group O, 6 different genotypes in children with blood group B and 2 different genotypes in children with blood group A (Table 5).

### Phylogenetic Analysis Revealed Unusually Large Genetic Diversity of Norovirus in Burkina Faso

**Figure 1:**
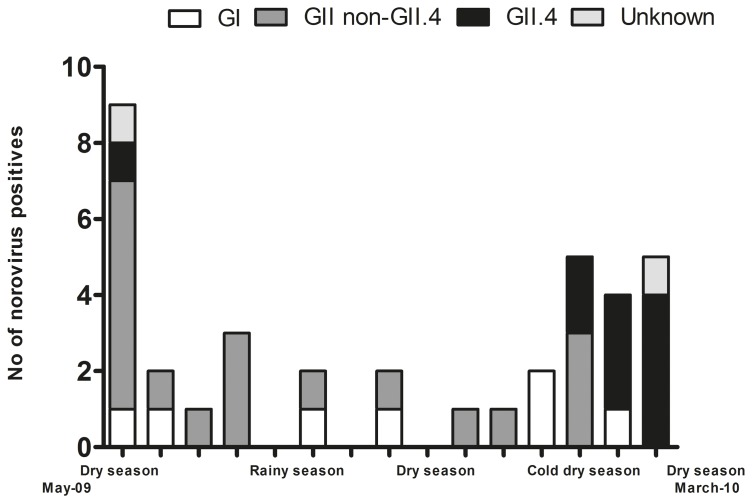
Temporal distribution of norovirus genogroups/genotypes in Ouagadougou, Burkina Faso from end of May 2009 to March 2010; each tick representing 3 weeks. Rainy season (June-September), cold dry season (December-February).

**Figure 2:**
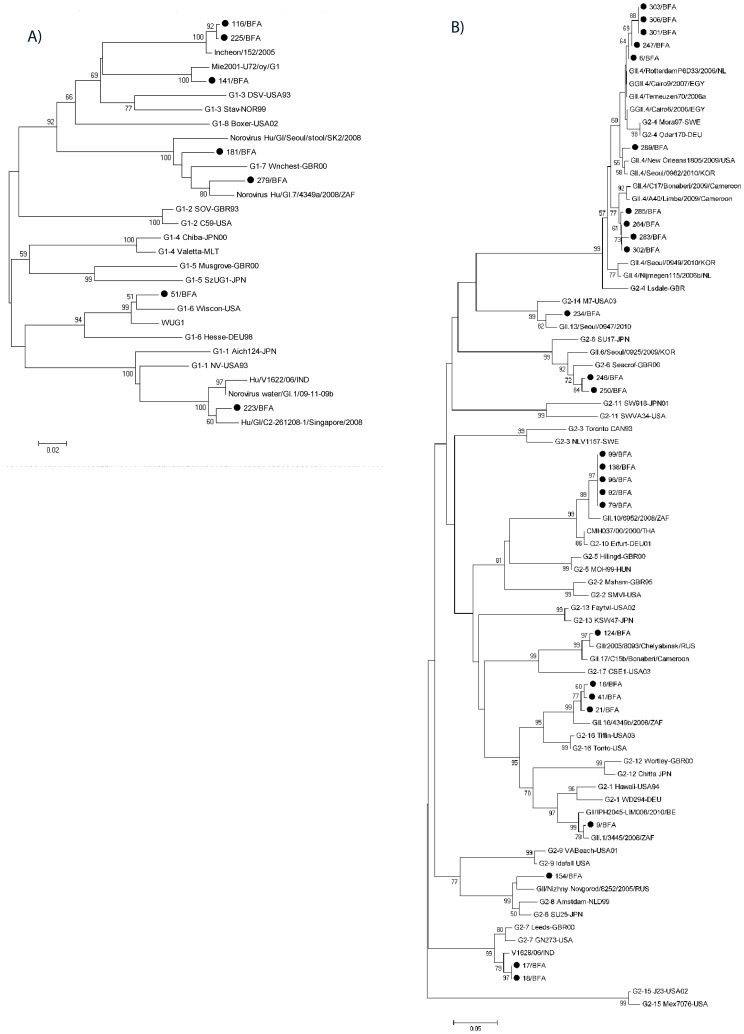
Phylogenetic analysis of the NS region (nt 1–301, ORF2) of NoV GI strains from Burkina Faso, May 2009–March 2010 with references strains from all known GI genotypes and most similar NoV strains detected in sub-Saharan Africa (panel A). Phylogenetic analysis of the NS region (nt 1–274) of NoV GII strains from Burkina Faso, May 2009–March 2010 with references strains from all known GII genotypes and most similar identities detected in sub-Saharan Africa (panel B). The Burkina Faso strains are marked with filled circles. Scale bar represents the number of substitutions per site and bootstrap values are shown at branch nodes (values of <50% are not shown).

A markedly large genetic diversity of circulating NoV strains was observed. Overall, the 37 NoV positive samples belonged to 14 genotypes: GI.1 (n = 1), GI.3 (n = 1), GI.6 (n = 1), GI.7 (n = 2), GII.1 (n = 1), GII.4 (n = 10), GII.6 (n = 3), GII.7 (n = 3), GII.8 (n = 1), GII.10 (n = 5), GII.14 (n = 1), GII.16 (n = 3) and GII.17 (n = 1) (Figures 1–2). Two GI samples, 116 and 225, belong to one as of yet undefined genotype (Figure 2A), whereas two GII samples could not be genotyped. The GII.4 genotypes (n = 10) could further be divided into three different variants; namely 2006a (n = 5; nt identity 98.5–99.6% to Terneuzen 2006a strain, Figure 2B), and two undefined variants (n = 1, isolate 289, nt identity 98.1% to New Orleans strain), and a variant (n = 4), most closely similar to GII.4 NoV strains detected in Cameroon (nt identity 97.4–98.1%) (Figure 2B). The two GI strains 116 and 225 that could not be assigned a genotype (86.1% nt identity to most similar reference sequence DSV [4]) were most similar to the Incheon 152 strain (98.5–98.9% nt identity) detected in South Korea in 2005.

The second most prevalent genotype in this study was GII.10 (n = 5), which is rarely described in literature, was similar to strain 6952, detected in hospitalized children in South Africa in 2008 (nt identity 97.8%). The third most prevalent genotypes were GII.6 (n = 3), GII.7 (n = 3), GII.16 (n = 3). The GII.6 strains were most similar to the Seacroft strain detected in UK in 1990 (nt identity 96.3–96.7%). The GII.7 strains were most similar to V1628 strain detected in a child with diarrhea in India 2006 (nt identity 98.5%) and the GII.16 strains were similar to strain 4349b (nt identity 96.7–97.0%), also detected in a hospitalized child in South Africa in 2008.

Most GII.4 strains occurred in the cold dry season, and all three GII.4 variants circulated simultaneously (Figures 1 and 2). At the end of the dry season before the start of the rainy season (May/June 2010) we observed a large NoV diversity with 6 different genotypes circulating at the same time (Figure 1). During the rainy season, few NoV were observed, and it was during this season that all genotype GII.10 (n = 5) NoVs were observed. The detection rate of NoV subsequently increases in the late dry season (from December 2009 and onwards until end of sample collection in middle of March 2010, Figure 1).

## Discussion

We have performed a molecular epidemiological study of NoV in pediatric diarrhea in Burkina Faso, and further correlated this with host susceptibility markers for symptomatic NoV infections. Surprisingly few reports on NoV are available from Africa and this is the first study to investigate the role of host genetic markers and susceptibility to NoV infection in an African setting.

The overall prevalence of NoV in pediatric diarrhea in Burkina Faso was 12% which is similar to studies from Nicaragua, Thailand, Spain and India [7], [34]–[36], but lower than other recent studies using similar PCR methodology for detection as in this study [1]. Plausibly, the NoV prevalence in Burkina Faso would have been higher if samples had been collected for a complete year, since the GII.4 genotypes appeared to be emerging at the end of the collection period (February-March 2010), which could indicate the start of the NoV “season”. Earlier reports indicate that the period with the highest NoV-induced diarrhea [37] (winter month in the Northern hemisphere) are mainly due to genotype GII.4. This would thus correspond to the late dry season in Burkina Faso. Interestingly, other studies from tropical climates in Central America have indicated the NoV season to correspond to the rainy season [7]. The lack of long term NoV surveillance in Sub-Saharan Africa, makes it difficult to assess to seasonality since most previous studies only collected samples during the dry season. However, one study from rural communities in Nigeria also report similar seasonality as described in this study [33].

Previously, the same stool samples used in this study have been screened for rotavirus and other enteropathogens [27], [28]. Comparing the seasonality, we observed that the NoV GII.4 “season” follow a few weeks after the peak of rotavirus induced diarrhea [27]; the same dual seasonality patterns with NoV prevalence increasing after rotavirus have also been observed in other countries [38], [39].

NoV infections were associated with fever (49%), vomiting (49%) and dehydration (62%). After excluding mixed infections from the analysis we observed similar clinical symptoms for “pure” NoV infections as compared to mixed infections with NoV and other enteropathogens. However, genotype GII.4 infections (n = 10), seemingly induced more severe symptoms with higher prevalence of vomiting, dehydration and longer duration of diarrhea. The distribution of concomitant infections in children with GII.4 and non-GII.4 genotypes was similar; with coinfection found in 60% of cases with GII.4 infection compared to 52% in the non-GII.4 group. The limited number of GII.4 genotypes, however, makes these results indicative. Similar observations have also been reported in previous studies [7], [10].

Children younger than one year of age in our cohort of children having diarrhea were more likely to be infected with NoV (p<0.05). The relatively high prevalence of NoV in the age group 0–6 months could be due to that mothers rarely exclusively breast feed in Burkina Faso [28]. No child older than 2 years of age in our cohort was infected with NoV, similar to findings reported from other developing countries [7], [39].

We investigated human HBGAs (ABH and Lewis a/b antigens) and susceptibility to NoV infections, for the first time in an African setting. The genetic polymorphisms mediating expression of human HBGAs varies greatly between different ethnicities and regions of the world [40], and most of the authentic studies have been performed in Caucasian populations with a limited number of NoV genotypes investigated [21], [41], [42]. For example, the Lewis-negative phenotype which is rare in Caucasian populations (∼6%; [43]), was highly prevalent in Burkina Faso (32%), a high prevalence has also been observed in Nicaragua and in small populations of African descendants in Brazil [44], [45]. The large variety of HBGA binding patterns to different NoV genotypes has been extensively investigated using virus-like particles (VLPs), and is believed important for understanding of NoV evolution and immune evasion [22], [23], [41]. However, there is limited saliva binding data with wild-type virus [20] and a rather few NoV genotypes have been investigated regarding HBGAs and susceptibility in natural infections.

This study confirms previous findings from Europe and the US that non-secretors are less susceptible to NoV infections, also in an African population and an extraordinary high diversity of circulating NoV genotypes, with two non-secretor infected with NoV of genotypes GII.7 and GII.4, respectively. Reports describing non-secretors infected with NoV are few in the literature but not unique [17], [23]–[25]. Interestingly, GII.7 VLPs have been shown to bind to saliva from non-secretors [22], which further strengthens the assumption that saliva binding studies can be used as a surrogate marker for susceptibility. Moreover, one non-secretor was infected with the globally dominant GII.4 genotype (strain 306; figure 2B); which has been rarely reported in literature [25]. Secretor-independent binding of recent GII.4 strains to the Lewis-fucose has been reported and suggested [46], [47]; thereby indicating that some GII.4 variants can infect non-secretors. Recently; Jin et al. [48]; described two outbreaks in China caused by GII.3 and GII.4 genotypes; where a few Lewis-positive non-secretors were also symptomatically infected. In this study; the secretor-negative child was also Lewis-negative; suggesting that the Lewis antigen was not required for infection. The two other GII.4 strains of the same variant (Figure 2B) infected secretors (1 Lewis-positive and 1 Lewis-negative). To our knowledge, this is the first report of a both secretor and Lewis-independent GII.4 strain; however due to the low numbers of samples this needs to be confirmed in more studies. The high diversity of NoV genotypes found in this studt makes it difficult to assess complete susceptibility patterns for individual genotypes due to limited number of samples for each genotype. Also; a potential weakness of the study design is that we do not have a true population control; only children with diarrhea were prospectively enrolled in the study and then stratified into NoV-positive and NoV-negatives. To our knowledge, no previous data of secretor or Lewis phenotypes exists from Burkina Faso. However; we believe the results from this study are useful for determining differences in host genetic susceptibility for different NoV strains.

While the Lewis b antigen is commonly used as a surrogate marker for determining secretor- positive status, and Lewis-negatives (Le^a−b−^) have antibodies to NoV [43], there is to our best knowledge, no *in vivo* information available if Lewis-negative individuals are susceptible to all genogroups or genotypes of NoV. An interesting observation from this study was not only that 32% of the investigated individuals were Lewis-negative, but also that the Lewis-negatives were equally susceptible to symptomatic GII infections as Le^a−b+^ individuals, and in contrast, no Lewis-negative child was infected with GI NoV (p = 0.07).79% of the Lewis-negative children were secretors and 21% were non-secretors; thus similar to the prevalence of secretor-phenotype in the entire cohort. Lewis-positive secretors (Le^a−b+^) have, besides the α1,2 linked fucose present on the galactose moiety on the H-type 1 antigen; also an α1,4-linked fucose to the GlcNAc moiety of the H-type 1 antigen thus making the Lewis b antigen. It is clear that presence of an α1,2 linked fucose is essential for susceptibility to a large variety of NoV genotypes [22], and presence of both α1,2 linked and α1,4-linked fucoses (Le^a−b+^) have been suggested to have a synergistic effect for some NoV strains [49]. As of yet however, due to the low prevalence of the Lewis-negative phenotype in most *in vivo* studies, it has not been clearly established whether the additional α1,4-linked fucose of Lewis-positive secretors could influence susceptibility. In Burkina Faso, nearly all (11/12) of the Lewis-negative children infected with GII NoVs were secretors; and no difference was observed in susceptibility compared to Lewis-positive secretors. These results indicate that for a large variety of genogroup II NoVs observed in this study; presence of the α1,4-linked fucose (Lewis) is not necessary for establishing symptomatic infection. The GI NoVs in this study however infected only Lewis-positive secretors, indicating that presence of both α1,2 linked and α1,4-linked fucose moieties could facilitate symptomatic infection for specific genotypes/genogroups. To confirm the identity of the Lewis-negative phenotype, the *FUT3* gene was sequenced from a subset of Lewis-negative children (data not shown). The unusual large number of GII genotypes identified in this study may be due to the high prevalence of Lewis-negatives, as 7/9 GII genotypes infected Lewis-negative children, including two genotypes only found in Lewis-negatives (Table 5). A possibility is that the high prevalence of Lewis-negative individuals contributes to viral evolution, although this is speculative. A most reasonable explanation why our observation not previously have been reported is the fact that most HBGA and NoV studies have been conducted on Caucasians [21], [50] with just a few percent Lewis-negatives [43].

NoV was observed less in blood group A secretor-positive children compared to other blood groups (p = 0.054) in the study population. We further observed that GI NoVs preferentially infected individuals with blood group O, whereas GII NoVs were more prevalent in individuals with blood group B. Blood group B has repeatedly been reported to mediate protection or partial protection towards GI NoVs, but not GII NoVs [17], [24], [51], which thus agree with the findings in this study. By comparing GII.4 with non-GII.4 genotypes, we observed that GII.4 NoVs preferentially infected individuals with blood group B whereas non-GII.4 genotypes were more likely to infect individuals of blood group O. Interestingly, Tan et al., [18] reported an outbreak of GII.4 viruses and found that blood group A was associated with increased susceptibility to infection which is in contrast to the findings of this study. This probably reflects changes in HBGA preferences between variants of GII.4 strains, which have previously been observed in binding studies and is believed important for GII.4 evolution [42].

The phylogenetic analyses demonstrated an unusually large genotype diversity including many uncommon genotypes. In total 14 genotypes and 3 different GII.4 variants were circulating in less than one year at the same location. Whether this large diversity is common, or indeed unique, for Africa needs to be confirmed in future studies. Of consideration is that children enrolled were those who sought medical assistance at the center, and thus the result could be biased towards more severe genotypes, and even still, 14 genotypes were detected. Of further note is that 11 different genotypes were found in secretor-positive children with blood group O; indicating that the secretor-positive phenotype with the absence of blood group antigens renders the individuals susceptible to a large variety of NoV genotypes of both genogroup I and II.

Of note is also that the Lewis-negative children were infected with a large variety of different GII genotypes. The differences in host genetic polymorphisms in HBGAs, particularly Lewis negatives, between African and Caucasians populations may lead to differences in circulating NoV strains, which may be using different HBGAs as cellular receptors.

Two GI NoV strains, 116 and 225 (Figure 2A) seemed to constitute a separate genotype cluster, although more sequence data from the entire capsid gene is needed for confirmation. One GII.4 variant was classified as 2006a, whereas the remaining two variants could not be assigned (max 98.1% nt identity of the NS region to known GII.4 variants and un-assignable by the NoV genotyping tool). Furthermore, many Burkina Faso NoV strains were of rare genotypes, which are seldom reported in the literature. Whether these genotypes are more common in the region investigated is difficult to establish due to the limited number of studies in Africa. In the light of the NoV vaccine trials that are being carried out [52], and the high mortality in developing countries [1], [53] it is urgent to determine which genotypes are circulating in Africa.

To conclude, we found a remarkably high genetic diversity of NoV in pediatric diarrhea in Burkina Faso. We further found that the Lewis b antigen is not required for symptomatic GII infection, but for GI infection. Another interesting observation regarding viral evolution was that 32% of the investigated were Lewis-negative (low prevalence in the Caucasian population) which certainly could contribute to NoV evolution and diversity in Africa.

## References

[1] Patel MM, Widdowson MA, Glass RI, Akazawa K, Vinje J, et al (2008) Systematic literature review of role of noroviruses in sporadic gastroenteritis. Emerg Infect Dis 14: 1224–1231. 18680645 10.3201/eid1408.071114 PMC2600393

[2] Bucardo F, Lindgren PE, Svensson L, Nordgren J (2011) Low prevalence of rotavirus and high prevalence of norovirus in hospital and community wastewater after introduction of rotavirus vaccine in Nicaragua. PLoS One 6: e25962. 22016794 10.1371/journal.pone.0025962 PMC3189239

[3] Puustinen L, Blazevic V, Salminen M, Hamalainen M, Rasanen S, et al (2011) Noroviruses as a major cause of acute gastroenteritis in children in Finland, 2009–2010. Scand J Infect Dis 43: 804–808. 21696253 10.3109/00365548.2011.588610

[4] Zheng DP, Ando T, Fankhauser RL, Beard RS, Glass RI, et al (2006) Norovirus classification and proposed strain nomenclature. Virology 346: 312–323. 16343580 10.1016/j.virol.2005.11.015

[5] Wang QH, Han MG, Cheetham S, Souza M, Funk JA, et al (2005) Porcine noroviruses related to human noroviruses. Emerg Infect Dis 11: 1874–1881. 16485473 10.3201/eid1112.050485 PMC3367634

[6] Mesquita JR, Barclay L, Nascimento MS, Vinje J (2010) Novel norovirus in dogs with diarrhea. Emerg Infect Dis 16: 980–982. 20507751 10.3201/eid1606.091861 PMC3086253

[7] Bucardo F, Nordgren J, Carlsson B, Paniagua M, Lindgren PE, et al (2008) Pediatric norovirus diarrhea in Nicaragua. J Clin Microbiol 46: 2573–2580. 18562593 10.1128/JCM.00505-08 PMC2519475

[8] Siebenga JJ, Vennema H, Zheng DP, Vinje J, Lee BE, et al (2009) Norovirus illness is a global problem: emergence and spread of norovirus GII.4 variants, 2001–2007. J Infect Dis 200: 802–812. 19627248 10.1086/605127

[9] Bucardo F, Nordgren J, Carlsson B, Kindberg E, Paniagua M, et al (2010) Asymptomatic norovirus infections in Nicaraguan children and its association with viral properties and histo-blood group antigens. Pediatr Infect Dis J 29: 934–939. 20657344 10.1097/INF.0b013e3181ed9f2f

[10] Desai R, Hembree CD, Handel A, Matthews JE, Dickey BW, et al (2012) Severe outcomes are associated with genogroup 2 genotype 4 norovirus outbreaks: A systematic literature review. Clin Infect Dis 55: 189–193. 22491335 10.1093/cid/cis372 PMC3491774

[11] Ayukekbong J, Lindh M, Nenonen N, Tah F, Nkuo-Akenji T, et al (2011) Enteric viruses in healthy children in Cameroon: viral load and genotyping of norovirus strains. J Med Virol 83: 2135–2142. 22012721 10.1002/jmv.22243

[12] Mans J, de Villiers JC, du Plessis NM, Avenant T, Taylor MB (2010) Emerging norovirus GII.4 2008 variant detected in hospitalised paediatric patients in South Africa. J Clin Virol 49: 258–264. 20869912 10.1016/j.jcv.2010.08.011

[13] Mattison K, Sebunya TK, Shukla A, Noliwe LN, Bidawid S (2010) Molecular detection and characterization of noroviruses from children in Botswana. J Med Virol 82: 321–324. 20029818 10.1002/jmv.21682

[14] Dove W, Cunliffe NA, Gondwe JS, Broadhead RL, Molyneux ME, et al (2005) Detection and characterization of human caliciviruses in hospitalized children with acute gastroenteritis in Blantyre, Malawi. J Med Virol 77: 522–527. 16254959 10.1002/jmv.20488

[15] Armah GE, Gallimore CI, Binka FN, Asmah RH, Green J, et al (2006) Characterisation of norovirus strains in rural Ghanaian children with acute diarrhoea. J Med Virol 78: 1480–1485. 16998875 10.1002/jmv.20722

[16] Kindberg E, Akerlind B, Johnsen C, Knudsen JD, Heltberg O, et al (2007) Host genetic resistance to symptomatic norovirus (GGII.4) infections in Denmark. J Clin Microbiol 45: 2720–2722. 17537929 10.1128/JCM.00162-07 PMC1951234

[17] Nordgren J, Kindberg E, Lindgren PE, Matussek A, Svensson L (2010) Norovirus gastroenteritis outbreak with a secretor-independent susceptibility pattern, Sweden. Emerg Infect Dis 16: 81–87. 20031047 10.3201/eid1601.090633 PMC2874438

[18] Tan M, Jin M, Xie H, Duan Z, Jiang X, et al (2008) Outbreak studies of a GII-3 and a GII-4 norovirus revealed an association between HBGA phenotypes and viral infection. J Med Virol 80: 1296–1301. 18461617 10.1002/jmv.21200

[19] Lindesmith L, Moe C, Marionneau S, Ruvoen N, Jiang X, et al (2003) Human susceptibility and resistance to Norwalk virus infection. Nat Med 9: 548–553. 12692541 10.1038/nm860

[20] Thorven M, Grahn A, Hedlund KO, Johansson H, Wahlfrid C, et al (2005) A homozygous nonsense mutation (428G–>A) in the human secretor (FUT2) gene provides resistance to symptomatic norovirus (GGII) infections. J Virol 79: 15351–15355. 16306606 10.1128/JVI.79.24.15351-15355.2005 PMC1315998

[21] Rydell GE, Kindberg E, Larson G, Svensson L (2011) Susceptibility to winter vomiting disease: a sweet matter. Rev Med Virol 21: 370–382. 22025362 10.1002/rmv.704

[22] Shirato H, Ogawa S, Ito H, Sato T, Kameyama A, et al (2008) Noroviruses distinguish between type 1 and type 2 histo-blood group antigens for binding. J Virol 82: 10756–10767. 18701592 10.1128/JVI.00802-08 PMC2573190

[23] Lindesmith L, Moe C, Lependu J, Frelinger JA, Treanor J, et al (2005) Cellular and humoral immunity following Snow Mountain virus challenge. J Virol 79: 2900–2909. 15709009 10.1128/JVI.79.5.2900-2909.2005 PMC548455

[24] Rockx BH, Vennema H, Hoebe CJ, Duizer E, Koopmans MP (2005) Association of histo-blood group antigens and susceptibility to norovirus infections. J Infect Dis 191: 749–754. 15688291 10.1086/427779

[25] Carlsson B, Kindberg E, Buesa J, Rydell GE, Lidon MF, et al (2009) The G428A nonsense mutation in FUT2 provides strong but not absolute protection against symptomatic GII.4 Norovirus infection. PLoS One 4: e5593. 19440360 10.1371/journal.pone.0005593 PMC2680586

[26] Soejima M, Munkhtulga L, Iwamoto S, Koda Y (2009) Genetic variation of FUT3 in Ghanaians, Caucasians, and Mongolians. Transfusion 49: 959–966. 19175549 10.1111/j.1537-2995.2008.02069.x

[27] Nordgren J, Nitiema LW, Sharma S, Ouermi D, Traore AS, et al (2012) Emergence of Unusual G6P[6] Rotaviruses in Children, Burkina Faso, 2009–2010. Emerg Infect Dis 18: 589–597. 22469076 10.3201/eid1804.110973 PMC3309693

[28] Nitiema LW, Nordgren J, Ouermi D, Dianou D, Traore AS, et al (2011) Burden of rotavirus and other enteropathogens among children with diarrhea in Burkina Faso. Int J Infect Dis 15: e646–652. 21763172 10.1016/j.ijid.2011.05.009

[29] Wardlaw T, Salama P, Brocklehurst C, Chopra M, Mason E (2010) Diarrhoea: why children are still dying and what can be done. Lancet 375: 870–872. 19833382 10.1016/S0140-6736(09)61798-0

[30] Rydell GE, Nilsson J, Rodriguez-Diaz J, Ruvoen-Clouet N, Svensson L, et al (2009) Human noroviruses recognize sialyl Lewis x neoglycoprotein. Glycobiology 19: 309–320. 19054801 10.1093/glycob/cwn139

[31] Kageyama T, Kojima S, Shinohara M, Uchida K, Fukushi S, et al (2003) Broadly reactive and highly sensitive assay for Norwalk-like viruses based on real-time quantitative reverse transcription-PCR. J Clin Microbiol 41: 1548–1557. 12682144 10.1128/JCM.41.4.1548-1557.2003 PMC153860

[32] Nordgren J, Bucardo F, Dienus O, Svensson L, Lindgren PE (2008) Novel light-upon-extension real-time PCR assays for detection and quantification of genogroup I and II noroviruses in clinical specimens. J Clin Microbiol 46: 164–170. 17959761 10.1128/JCM.01316-07 PMC2224280

[33] Japhet MO, Adesina OA, Famurewa O, Svensson L, Nordgren J (2012) Molecular Epidemiology of Rotavirus and Norovirus in Ile-Ife, Nigeria: High Prevalence of G12P[8] Rotavirus Strains and Detection of a Rare Norovirus Genotype. J Med Virol 84: 1489–1496. 22825829 10.1002/jmv.23343

[34] Khamrin P, Maneekarn N, Peerakome S, Tonusin S, Malasao R, et al (2007) Genetic diversity of noroviruses and sapoviruses in children hospitalized with acute gastroenteritis in Chiang Mai, Thailand. J Med Virol 79: 1921–1926. 17935183 10.1002/jmv.21004

[35] Sanchez-Fauquier A, Montero V, Moreno S, Sole M, Colomina J, et al (2006) Human rotavirus G9 and G3 as major cause of diarrhea in hospitalized children, Spain. Emerg Infect Dis 12: 1536–1541. 17176568 10.3201/eid1210.060384 PMC3290946

[36] Monica B, Ramani S, Banerjee I, Primrose B, Iturriza-Gomara M, et al (2007) Human caliciviruses in symptomatic and asymptomatic infections in children in Vellore, South India. J Med Virol 79: 544–551. 17385696 10.1002/jmv.20862 PMC2473265

[37] Kroneman A, Verhoef L, Harris J, Vennema H, Duizer E, et al (2008) Analysis of integrated virological and epidemiological reports of norovirus outbreaks collected within the Foodborne Viruses in Europe network from 1 July 2001 to 30 June 2006. J Clin Microbiol 46: 2959–2965. 18650354 10.1128/JCM.00499-08 PMC2546741

[38] Tran A, Talmud D, Lejeune B, Jovenin N, Renois F, et al (2010) Prevalence of rotavirus, adenovirus, norovirus, and astrovirus infections and coinfections among hospitalized children in northern France. J Clin Microbiol 48: 1943–1946. 20305010 10.1128/JCM.02181-09 PMC2863921

[39] Abugalia M, Cuevas L, Kirby A, Dove W, Nakagomi O, et al (2011) Clinical features and molecular epidemiology of rotavirus and norovirus infections in Libyan children. J Med Virol 83: 1849–1856. 21837804 10.1002/jmv.22141

[40] Koda Y, Soejima M, Kimura H (2001) The polymorphisms of fucosyltransferases. Leg Med (Tokyo) 3: 2–14. 12935727 10.1016/s1344-6223(01)00005-0

[41] Donaldson EF, Lindesmith LC, Lobue AD, Baric RS (2010) Viral shape-shifting: norovirus evasion of the human immune system. Nat Rev Microbiol 8: 231–241. 20125087 10.1038/nrmicro2296 PMC7097584

[42] Lindesmith LC, Donaldson EF, Lobue AD, Cannon JL, Zheng DP, et al (2008) Mechanisms of GII.4 norovirus persistence in human populations. PLoS Med 5: e31. 18271619 10.1371/journal.pmed.0050031 PMC2235898

[43] Larsson MM, Rydell GE, Grahn A, Rodriguez-Diaz J, Akerlind B, et al (2006) Antibody prevalence and titer to norovirus (genogroup II) correlate with secretor (FUT2) but not with ABO phenotype or Lewis (FUT3) genotype. J Infect Dis 194: 1422–1427. 17054072 10.1086/508430

[44] Bucardo F, Kindberg E, Paniagua M, Grahn A, Larson G, et al (2009) Genetic susceptibility to symptomatic norovirus infection in Nicaragua. J Med Virol 81: 728–735. 19235844 10.1002/jmv.21426

[45] Corvelo TCO, Aguiar DCF, Sagica FES (2002) The expression of ABH and Lewis antigens in Brazilian semi-isolated Black communities. Genet Mol Biol 25: 259–263.

[46] de Rougemont A, Ruvoen-Clouet N, Simon B, Estienney M, Elie-Caille C, et al (2011) Qualitative and quantitative analysis of the binding of GII.4 norovirus variants onto human blood group antigens. J Virol 85: 4057–4070. 21345963 10.1128/JVI.02077-10 PMC3126233

[47] Nasir W, Frank M, Koppisetty CA, Larson G, Nyholm PG (2012) Lewis histo-blood group alpha1,3/alpha1,4 fucose residues may both mediate binding to GII.4 noroviruses. Glycobiology 22: 1163–1172. 22589081 10.1093/glycob/cws084

[48] Jin M, He Y, Li H, Huang P, Zhong W, et al (2013) Two gastroenteritis outbreaks caused by GII Noroviruses: host susceptibility and HBGA phenotypes. PLoS One 8: e58605. 23472212 10.1371/journal.pone.0058605 PMC3589376

[49] Huang P, Farkas T, Zhong W, Tan M, Thornton S, et al (2005) Norovirus and histo-blood group antigens: demonstration of a wide spectrum of strain specificities and classification of two major binding groups among multiple binding patterns. J Virol 79: 6714–6722. 15890909 10.1128/JVI.79.11.6714-6722.2005 PMC1112114

[50] Kindberg E, Svensson L (2009) Genetic basis of host resistance to norovirus infection. Future virology 4: 369–382.

[51] Hutson AM, Atmar RL, Graham DY, Estes MK (2002) Norwalk virus infection and disease is associated with ABO histo-blood group type. J Infect Dis 185: 1335–1337. 12001052 10.1086/339883

[52] Parra GI, Bok K, Taylor R, Haynes JR, Sosnovtsev SV, et al (2012) Immunogenicity and specificity of norovirus Consensus GII.4 virus-like particles in monovalent and bivalent vaccine formulations. Vaccine 30: 3580–3586. 22469864 10.1016/j.vaccine.2012.03.050 PMC3359014

[53] Atmar RL, Bernstein DI, Harro CD, Al-Ibrahim MS, Chen WH, et al (2011) Norovirus vaccine against experimental human Norwalk Virus illness. N Engl J Med 365: 2178–2187. 22150036 10.1056/NEJMoa1101245 PMC3761795

